# Cercozoan diversity of spring barley grown in the field is strongly plant compartment specific

**DOI:** 10.3389/frmbi.2024.1352566

**Published:** 2024-02-08

**Authors:** Julia Sacharow, Stefan Ratering, Santiago Quiroga, Rita Geißler-Plaum, Bellinda Schneider, Alessandra Österreicher Cunha-Dupont, Sylvia Schnell

**Affiliations:** Professorship of General and Soil Microbiology, Institute of Applied Microbiology, Research Centre for Biosystems, Land Use and Nutrition, Justus-Liebig-University Giessen, Giessen, Germany

**Keywords:** *Hordeum vulgare*, soil cercozoa, leaf cercozoa, root cercozoa, holobiome

## Abstract

Protists are an important part of the plant holobiome and influence plant growth and pathogenic pressure as consumers. *Hordeum vulgare* is one of the most economically important crops worldwide, and its yield depends on optimal environmental plant-growth conditions and pathogen defense. This study aimed to analyse the natural compositions of the cercozoan diversity, one of the most important and dominant protist phyla, of spring barley at different developmental stages, from different plant compartments over two years. *Hordeum vulgare* bulk soil samples were taken before seeding and after harvest on an organic farming field. Bulk soil, rhizosphere soil, roots and leaves were sampled at the flowering and ripening stages, and analysed with cercozoan-specific primers. Results showed a clear dominance of the families Sandonidae, Allapsidae, Cercomonadidae, Rhogostomidae and the order Glissomonadida in all sample types. Separated analyses of root, leaf and soil samples showed that members of the family Sandonidae were strongly enriched in leaf samples, while members of the Allapsidae family were enriched in the roots. No compositional differences were detected between the different plant developmental stages, except for the beta diversity of the leaf samples at the flowering and ripening stages. It can be concluded that the cercozoan diversity of spring barley is primarily affected by the plant compartment and not by the plant developmental stage. Further studies are needed to analyze the cercozoan community in greater taxonomic depth and to target their ecological function.

## Introduction

Protists are an essential component of the biodiversity and ecosystem functioning of soils. They have diverse feeding behaviours consisting of bacterivores, fungivores, omnivores, mixotrophs and phototrophs protists but with specific prey spectra (Geisen, 2016; Dumack et al., 2019; Asiloglu et al., 2020). Their influence on the soil microbial community via consumption also affects the performance of the surrounding plants through plant growth promotion and plant health improvement (Jentschke et al., 1995; Krome et al., 2010; Weidner et al., 2017; Bahroun et al., 2021). Phagotrophic protists, for example, were shown to act as top-down controller of plant pathogens, and the ciliate *Colpoda cucullus* increased the dry matter content of maize (Wu et al., 2022; Zhang et al., 2022). Despite their diversity, ecological importance as predators of the soil microbiome, and bio-indicators of soil quality (Zhao et al., 2019), they are under-researched in comparison to bacteria and fungi, although they are also good candidates for use in biological crop protection (Sacharow et al., 2023).

To understand the whole plant-microbe-soil system it is important to analyse protist patterns on plants: they were shown to be strongly shaped by plant biomass, soil pH-value and moisture (Öztoprak et al., 2020), whereas land use is controversial. While Glaser et al. (2015) showed only a small effect of land use on protist communities, Geisen et al. (2015) showed that the protist community was strongly influenced by land use: where forest and grassland soils were dominated by Rhizaria and Amoebozoa, peat soils were dominated by Alveolata. Santos et al. (2020) showed that protist trophic groups were also affected by the land use intensity. Analysis of the protist community composition of switch grass revealed a lower diversity in the rhizosphere soil than in the bulk soil. The protist composition of the rhizosphere soil was mainly controlled by dispersal constraints and plant selection (Guo et al., 2018; Ceja-Navarro et al., 2021). Similarly, richness decreased with increasing soil depth, as well as with the use of chemical fungicides (Guo et al., 2018; Degrune et al., 2019). Glyphosate treatment of barley leaves altered protist communities on roots and their interactions with the surrounding prey (Imparato et al., 2016). A comparison between rhizosphere and bulk soil after the application of chemical fungicides revealed an increased amount of Alveolata and Amoebozoa (Guo et al., 2018). Protist community composition is also strongly influenced by fertilisation. In comparison to other microorganisms in the soil, nitrogen fertilisation had a higher impact on protists than on bacteria and fungi (Zhao et al., 2019). Krashevska et al. (2014) also showed that amoeba communities are primarily structured by abiotic factors and antagonistic interactions, rather than by prey availability.

The phylum Cercozoa (Cavalier-Smith and Chao, 2003) is a large group of free-living protists, which are an important part of the soil ecosystem (Bass and Cavalier-Smith, 2004). A cloning-based analysis of Brassicaceae leaves showed a highly diverse leaf-associated Cercozoa community composed of bacterivores, plant pathogens and endophytes (Ploch et al., 2016). Flues et al. (2018) discovered that the phyllosphere and rhizosphere of various plants are dominated by the genera *Cercomonas*, *Neocercomonas* and *Paracercomonas*. They found differences in diversity between the phyllosphere and the rhizosphere, but no differences between plant species. In contrast, Walden et al. (2021) demonstrated that the Cercozoa communities of some plants were specific to plant species, and their diversity was also seasonally influenced, changing from spring to autumn. Furthermore, they were sensitive to invasions of the ecosystem engineer earthworm, changing to an earthworm-associated community of Cercozoa (Dumack et al., 2022). Different environments are dominated by different groups of Cercozoa. For example, grasslands were dominated by Sarcomonadea (69%) and dunes by Thecofilosea (43%). However, the families Sandonidae, Allapsidae, and Rhogostomidae prevailed in both environments (Roshan et al., 2021). An analysis of agricultural and forest soils used for wheat plants showed that the cercozoan rhizosphere community was influenced by soil type, as well as the genotype of the wheat plant, and the soils were dominated by Sarcomonadea (42.3%), followed by Thecofilosea (27.1%) and Imbricatea (19.2%). The agricultural soil was dominated by the families Limnofilidae, Protaspididae, Thaumatomonadidae and unclassified Cryomonadida, while the forest soil was dominated by Rhogostomidae, Mesofilidae, unclassified Cercozoa, unclassified Imbricatea and unclassified Tectofilosida (Rossmann et al., 2020).

*Hordeum vulgare*, first cultivated around 7800-7500 B.C., is currently one of the most economically important crops besides wheat, rice and maize and is used in the production of alcoholic beverages and animal feed (Nesbitt and Samuel, 1996; Chełkowski et al., 2003). The aim of this project was to analyse the Cercozoan communities’ composition of this important crop from different sampling materials, at different growing stages, for two seasons. The results of the analysis will give an overview of the Cercozoan patterns of the plant and can help to understand the whole plant-microbe-soil system of barley.

## Materials and methods

### Seed preparation

*Hordeum vulgare* ODILIA (Öko Korn Nord, Germany) seeds were covered with a gum arabic and talc mixture before sowing. Gum arabic 25% (Roth, Germany) and talc powder (Roth, Germany) were autoclaved individually. The gum arabic was adjusted (Mettler-Toledo, Germany) to pH 7, mixed in a 1:1 ratio with MgSO_4_ (Roth, Germany) and shaken at 125 RPM on an orbital shaker (PSU-20i, Bio San, Latvia) at 20°C for 20 min. The mixture was then slowly added to the seeds until they were covered completely. Finally, talc powder was added and the seeds mixed (Kloepper, 1981). This study was part of a larger experimental project (https://www.bonares.de/bread-and-beer) with different seed inoculations in which the mixture of gum arabic and talc was used for the control plants, while plant growth bacteria were added to other treatments not studied here.

### Sampling fields on the Gladbacherhof

Fields on the Hessian State Domain Gladbacherhof (50° 23' N, 8° 15' E) of the Justus-Liebig-University Giessen were used for the cultivation of the plants. Seeds were sown in 1.5 x 5 m plots at four randomly selected points in the field using a tractor and seed drill. The plots for this experiment were surrounded by plots from the other project experiments with different seed inoculations. *Hordeum vulgare* ODILIA was sown in April 2021 and March 2022 and weeds were removed by hand when the plants were small.

### Sampling of plant material

Soil and plants (when applicable) were sampled before seeding, at the flowering and ripening stages, and after harvesting. Before seeding, 30 cm of the top soil were sampled with a Dutch auger. The samples were scratched into new plastic bags, transported to the laboratory on ice, sieved through a 2 mm size sieve, and stored in tubes (Sarstedt, Germany) at -20°C. At flowering, randomly selected plants were dug up, placed in new plastic bags and transported on ice to the laboratory. The bulk soil and the rhizosphere soil were separated by shaking. Bulk and rhizosphere soil were sieved to a size of 2 mm and stored in tubes at -20°C. Furthermore, soil-free parts of the roots and leaves were cut off with sterile scissors. The plant material was stored in tubes at -20°C. At the ripening, samples were taken in the same way as at flowering and after harvesting, the soil was sampled the same way as before the seeding.

### Analysis of chemical soil parameters

The bulk soil samples from before the seeding were analysed for NH_4_^+^, NO_3_^−^ and C:N. Ammonia content was determined by the method of Kandeler and Gerber (1988) after extraction with 1 M KCl. The nitrate was extracted from the soil according to the method in Cardinale et al. (2020) and nitrate content was determined with ion chromatography (Bak et al., 1991). For the C:N ratio measurement the soil was dried at 105°C for 24 h in a drying cabinet (ULE 500, Memmert, Germany) and milled in a Retsch mill (MM400, Retsch GmbH, Germany). In detail, the samples were placed in 2 ml tubes (Sarstedt, Germany), small iron balls were added and the tubes were shaken for 2 min at 30 s^-1^. Milled soil (35 mg) was filled in small tin boats (4 x 4 x 11 mm, Elementar Analysensysteme, Germany), filled boats were pressed together and measured with an elemental analyser (Unicube, Elementar, Germany).

### DNA extraction

The roots were washed by adding sterilized pure water in 2 ml tubes (Sarstedt, Germany) and shaken for 30 s. The washed roots were transferred into new 2 ml tubes and washed again until no further soil remains were observed in the water. Leaf and washed root material were cut into pieces with sterile scissors and crushed in liquid nitrogen using a sterile pestle and mortar. For the DNA extraction 100 - 150 mg leaf material, 150 – 250 mg root material and 300 – 350 mg soil material were used according to the protocol of Abdullaeva et al. (2021). After the extraction, the DNA was stored at -20°C.

### Amplicon library preparation and ion torrent sequencing

The V4 variable region of the 18S rRNA gene from the leaf material, root material, rhizosphere and bulk soil was amplified in a semi-nested PCR to analyse the cercozoan diversity. In the first step, the primer pair S616F_Cerco (5’-TTAAAAAGCTCGTAGTTG-3’) and S616F_Eocer (5’-TTAAAAAGCGCGTAGTTG-3’) were used as forward primers and S963R_Cerco (5’-CAACTTTCGTTCTTGATTAAA-3’) as reverse primer. In the second step of the semi-nested PCR the same forward primers were used as before with the S947R_Cerco (5’-AAGAAGACATCCTTGGTG-3’) reverse primer and barcodes (Fiore-Donno et al., 2017). The PCR reaction for the amplification (MycyclerTM, Bio-Rad, USA) was prepared as described by Fiore-Donno et al. (2017) with 2 µl of DNA template for both steps of the semi-nested PCR and an optimised thermal program (**Table 1**). Further steps of the Ion Torrent metabarcoding were done as described by Abdullaeva et al. (2021) with a final pool concentration of 300 pM.

**Table 1:** Optimised touchdown PCR program according to Fiore-Donno et al. (2017) for the amplification of the V4 variable region of the 18S rRNA gene with the cercozoan specific primer pairs S616F_Cerco, S616F_Eocer and S963R_Cerco as well as S616F_Cerco, S616F_Eocer and S947R_Cerco.

Step – First PCR	Temperature – First PCR	Time – First PCR	
1.	95°C	3 min	Steps 2-4 repeated for 24 times.
2.	98°C	20 s	
3.	50°C	20 s	
4.	72°C	30 s	
5.	72°C	5 min	
		
Step – Second PCR	Temperature – Second PCR	Time – Second PCR	
1.	95°C	3 min	Steps 2–4 repeated for 5 times with atemperature dec- rement of 1°Cper cycle in step 3.Steps 5-6 repeated for 19 times.
2.	98°C	20 s	
3.	70°C	30 s	
4.	72°C	30 s	
5.	98°C	20 s	
6.	65°C	30 s	
7.	72°C	30 s	
8.	72°C	5 min	

### Data analysis

The raw Ion Torrent sequences were processed with the bioinformatic pipeline QIIME2 version 2021.2 (Bolyen et al., 2019). The QIIME2 cutadapt plugin (Martin, 2011) was used for demultiplexing of the gene sequences and QIIME2 plugin DADA2 (Callahan et al., 2017) was used for quality control, filtering, chimera identification, denoising, summarizing the amplicon sequence variation (ASV) table, which records the number of observations of each exact ASV in each sample. The taxonomy was assigned by the q2-feature-classifier (Bokulich et al., 2018) trained on the PR2 database 4.14.0 (Guillou et al., 2013). The ASVs that were identified as non-cercozoan were removed.

### Statistical analysis

Data analysis of the ASV table was performed with QIIME2 version 2021.2, R-Studio version 4.2.3 (R Core Team, 2020), the phyloseq 1.28.0 package (McMurdie & Holmes, 2014) and the qiime2r 0.99.6 package (Bisanz, 2018). Alpha-diversity (R-Studio version 4.2.3) was determined by the mean value from the ASV table with observed richness, Shannon diversity index (Shannon and Weaver, 1964) and Fisher diversity index (Fisher et al., 1943) after rarefaction. The significant differences were determined with the Wilcox test (Wilcoxon, 1945) and the Holm correction method (Holm, 1979) through 999 permutations. For the plots ggplot2 3.4.2 (Wickham, 2016) was used. Beta-diversity (QIIME2 version 2021.2 and R-Studio version 4.2.3) was obtained with Aitchison principal component analysis (PCA) as well as Robust Aitchison principal component analysis (RPCA) with a centred log-ratio transform (CLR) and a robust centred log-ratio transform (RCLR) (DEICODE, Martino et al., 2019). The significant differences were determined by a Permutational Multivariate Analysis of Variance (PERMANOVA) using the Adonis method (Anderson, 2001) with a Benjamini–Hochberg (Benjamini and Hochberg, 1995) correction and employing 999 permutations. The cercozoan ASVs from the underground samples (bulk soil, rhizosphere soil and roots) were pre-processed (R-Studio version 4.2.3) as to keep all ASVs present in a minimum of two samples and a minimum of five reads per ASV. The cercozoan ASVs from the leaf samples were not pre-processed. For the compositional data analysis R-Studio version 4.2.3 and the package ALDEx2 1.22.0 (Fernandes et al., 2013) were used. An ALDEx2 test was done by performing a centred log ratio (clr) transformation (Aitchison, 1982; Aitchison, 1986) using as denominator the geometric mean abundance of all features and 128 Monte-Carlo instances. Then a Welch’s t-test with a Benjamini–Hochberg (Benjamini & Hochberg, 1995) correction was carried out. The phylogenetic tree was calculated with QIIME2 version 2021.2 based on the maximum likelihood method and adapted with R-Studio version 4.2.3, with the packages ape 5.7.1 (Paradis et al., 2004) and ggtree 3.8.2 (Guangchuang, 2022). For the summary figure illustrations of the University of Maryland Center for Environmental Science Integration and Application Network (https://ian.umces.edu/media-library/) were used and merged.

## Results

### Chemical analysis

The chemical analysis of the bulk soil before seeding of the two seasons of spring barley showed similar ammonium mean concentrations, similar mean C:N ratios and similar mean soil temperatures, but different nitrate mean concentrations (**Table 2**).

**Table 2:** Mean values of the chemical analysis of the bulk soil before seeding of the two seasons of spring barley.

Spring barley season	NH_4_^+^ [µmol g^−1^ DW soil]	NO_3_^-^ [µmol g^−1^ DW soil]	C:N ratio	Soil temperature [°C]
First season 2021	317.02	346.11	9.14	5.1
Second season 2022	331.36	869.25	8.06	6.3

DW, Dry weight.

### Ion torrent sequencing and pre-processing

A total of 640.438 and 3.840.352 raw sequences (two seasons collapsed, see **Supplementary Figure 1** for comparison of the two seasons) were obtained for leaf samples and underground material (roots, bulk soil, rhizosphere soil), respectively. The sequence counts ranged from 12.521 to 103.274 for the leaf material and from 34.658 to 113.092 for the underground material. After quality control, denoising, sequence dereplication and chimera filtering, 108.622 sequences were removed from the leaf material and 851.482 sequences from the underground material. There were 531.816 remaining sequences for the leaf material and 2.988.870 remaining sequences for the underground material. They were grouped in 3619 cercozoan ASVs for the underground material, pre-processed (before performing differential abundance analysis to handle different types of zeros like structural zero, outlier zero, and sampling zero) to 1868 remaining ASVs. Sequences were grouped into 389 ASVs for the leaf material. Pre-processing the leaf material resulted in 63 remaining ASVs only, this step was therefore not carried out.

### Taxonomic diversity

**Figure 1:**
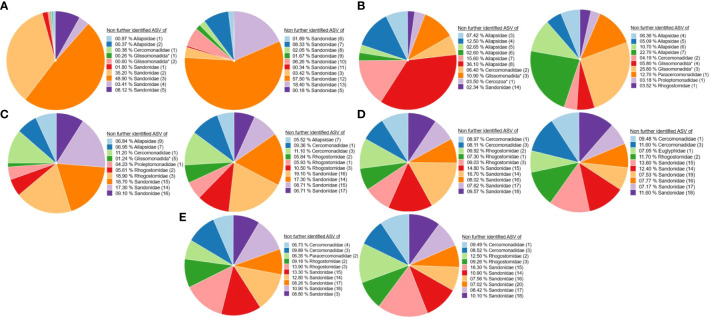
Ten most abundant (taxonomically not further determined than the family level) ASVs of cercozoan families. **(A)** Leaf samples at flowering (left) and ripening (right) stages. **(B)** Root samples at flowering (left) and ripening (right) stages. **(C)** Rhizosphere soil samples at flowering (left) and ripening (right) stages. **(D)** Bulk soil samples at flowering (left) and ripening (right) stages. **(E)** Bulk soil samples before seeding (left) and after harvesting (right). * Taxonomically not determined to family level. The number in brackets is the corresponding number of the ASV.

The ten most abundant ASVs in the leaf, root and soil samples could not be taxonomically determined further than the family level (hereafter: unidentified ASVs). These ASVs are marked with numbers to distinguish them. They belonged to the families Sandonidae, Allapsidae, Cercomonadidae, Glissomonadida and Rhogostomidae (**Figure 1**). Leaf samples were dominated by several unidentified ASVs of the Sandonidae family (**Figure 1A**), whereas the root samples were dominated by several unidentified ASVs of the Allapsidae family, as well as Cercomonadidae (2) and Glissomonadida (3 and 4) (**Figure 1B**). At the ripening stage, unidentified ASVs of Paracercomonadidae (1) and Proleptomonadidae (1) families appeared. All soil samples were dominated by several unidentified ASVs (**Figures 1C–E**). The rhizosphere soil was composed of unidentified ASVs belonging to the families Sandonidae, Allapsidae, one Cercomonadidae (1) ASV and Rhogostomidae (**Figure 1C**), whereas the bulk soil was dominated by unidentified ASVs of the families Sandonidae, Cercomonadidae and Rhogostomidae. At the ripening stage, the unidentified ASV of the Euglyphidae (1) family appeared and before seeding unidentified ASV of the family Paracercomonadidae (2) was present (**Figures 1D–E**). A summary overview of these results can be seen in **Supplementary Figure 2**.

### Alpha diversity

**Figure 2:**
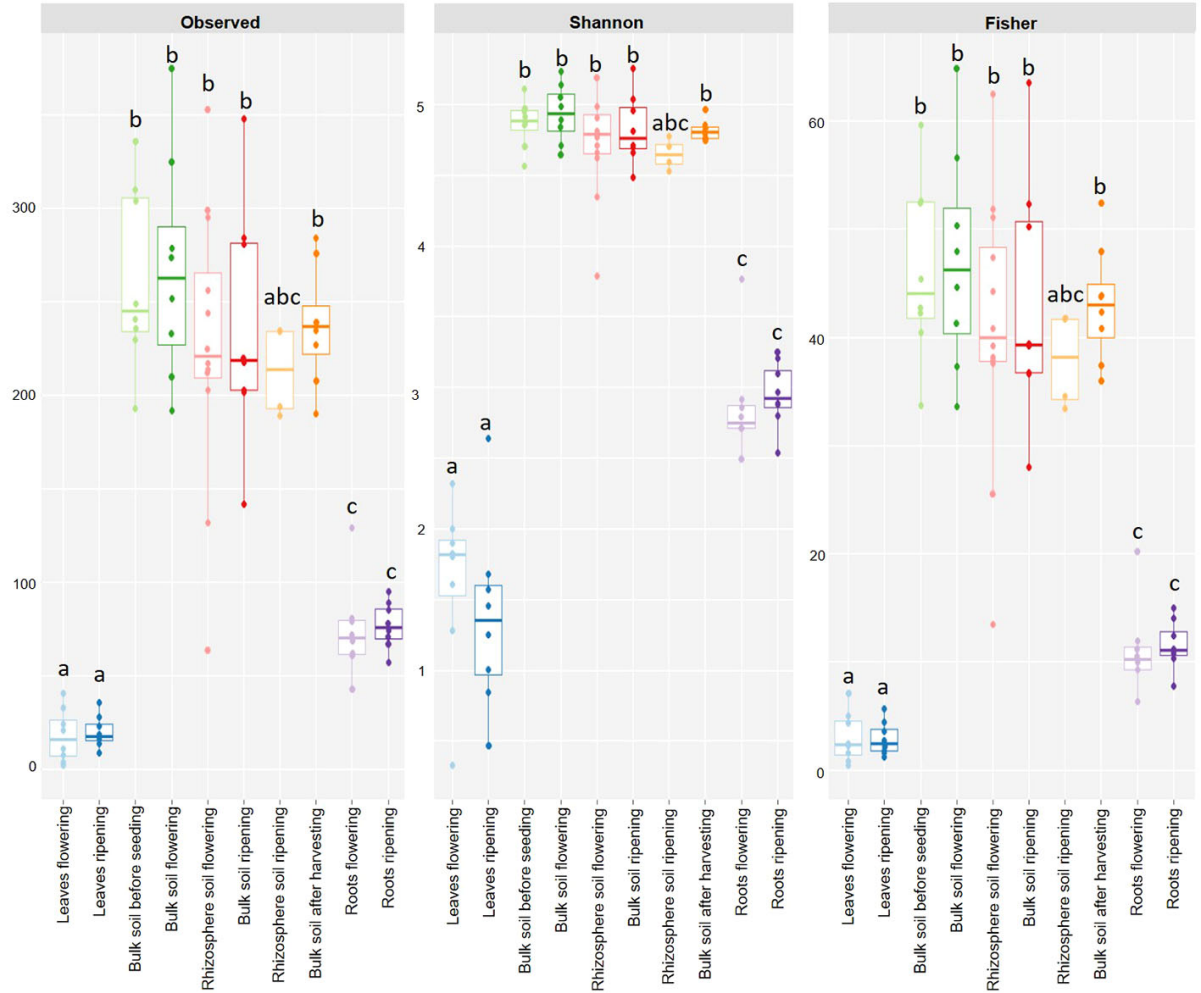
Alpha diversity measurements of leaf (blue), soil (green to orange) and root (purple) samples before seeding, at flowering, at ripening and after harvest with the observed ASVs, Shannon’s and Fisher’s diversity. The distinct letters delineate significantly diverse groups.

The observed ASV number, Shannon’s and Fischer’s diversity indices showed differences in the alpha diversity of the sampling material (**Figure 2**). There was a clear separation of the alpha diversity from the leaf material, from the soil material and the root material. There were no significant differences with the Wilcox test independent of the index considered between the leaf samples of the flowering and the ripening stages (*p*-value 1.00), as well as among the soil samples of the different sampling stages (*p*-values from 0.48 to 1.00), and the root samples of the different sampling stages (*p*-value 1.00). In contrast, significant differences between the different sampling materials could be detected. Alpha diversity of leaf and soil samples were significantly different independent of the index (*p*-values < 0.05). However, diversity indices of leaf material at flowering and ripening did not significantly differ from rhizosphere soil at ripening (*p*-values above 0.08). The same could be detected for the comparison of the root and soil material: no significant differences in alpha diversity could be detected between root material at flowering and ripening and rhizosphere soil at ripening (*p*-values above 0.08). Only significant differences could be detected, independent of the index (*p*-values < 0.05), of the two root samples and the other soil samples. Furthermore, only significant alpha diversity differences were found between the leaf samples and the root samples, independent of the index considered (*p*-values < 0.05). See the supplementary data for exact *p*-values (**Supplementary Tables 1–3**).

### Beta diversity

**Figure 3:**
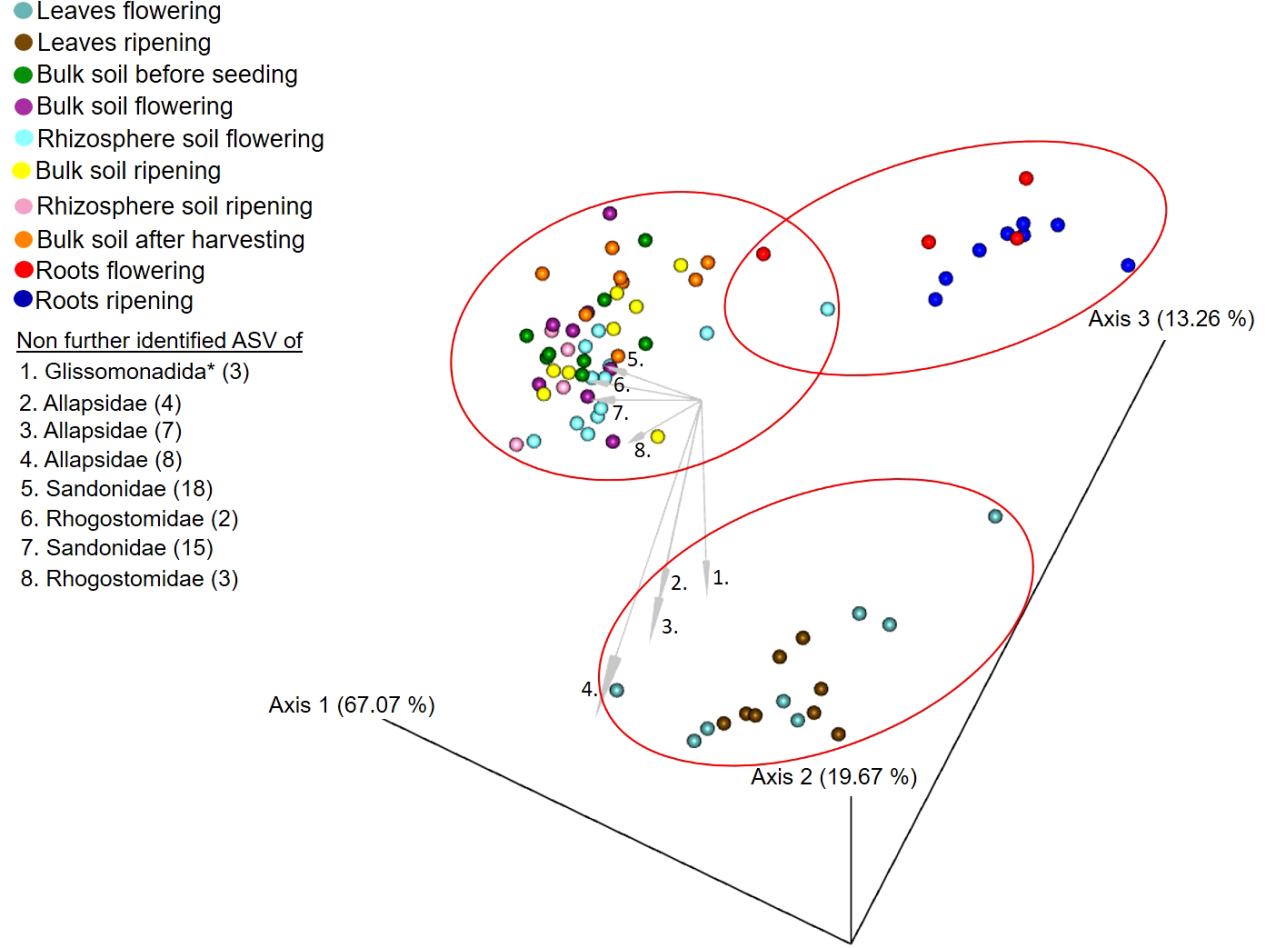
Robust principal component analysis plot of beta diversity measurement based on Aitchison distances of the leaf, soil and root samples before seeding, at flowering, at ripening and after the harvesting of the spring barley. Ellipses indicate groups of significantly different samples. Arrows indicate ASVs with the strongest influence on the beta diversity taxonomically not further determined than the family level. * Taxonomically not determined to family level. The number in brackets is the corresponding number of the ASV.

The RPCA of the beta diversity (Aitchison distances) showed a clear separation of leaf, soil and root samples (**Figure 3**). Each of them formed its own group with only a few overlapping points in between. PERMANOVA showed significant differences (*p*-value = 0.001) between all groups, and the pairwise PERMANOVA also showed several differences (**Supplementary Table 4**). Leaves’ community composition from the flowering stage was significantly different from that of leaves at the ripening stage (*p*-value 0.03). Furthermore, protist community composition of leaves from the flowering stage was significantly different in comparison to those from rhizosphere soil at flowering (*p*-value 0.04) and ripening (*p*-value 0.02), as well as those from root samples at both stages (*p*-values < 0.01). Leaf community composition at the flowering stage was not significantly different to the bulk soil community compositions before seeding, at the flowering stage, at the ripening stage and after harvest (*p*-values > 0.13). In contrast to this, the leaf material of the ripening stage was significantly different to all soil samples and root samples (*p*-values < 0.01). As for the alpha diversity, beta diversity was not significantly different between soil samples (*p*-values > 0.07), except for the rhizosphere soil ones. The rhizosphere soil cercozoan community composition at the flowering stage was significantly different from the one from rhizosphere soil at ripening (*p*-value 0.02). Furthermore, beta diversity of soil samples was significantly different from that of root samples (*p*-values < 0.01), but root samples’ composition from the flowering and ripening stages was not significantly different from each other (*p*-value 0.53). See the supplementary data for exact *p*-values (**Supplementary Table 4**). In addition, **Figure 3** also shows represented as arrows that unidentified ASVs belonging to the families Allapsidae, Sandonidae, Rhogostomidae and one ASV of the order Glissomonadida (3) have the biggest influence on the beta diversity.

### Differential abundance

The MA plot and MW plot from root and leaf samples (**Supplementary Figure 3**) as well as the plots from soil and leaf samples (**Supplementary Figure 5**) showed an even distribution of differentially abundant ASVs in comparison to the mean (red dots), while the plots of the soil and root samples (**Supplementary Figure 4**) showed more red dots at the root than at the soil samples, indicating more abundant ASVs than the mean in the root samples.

**Figure 4:**
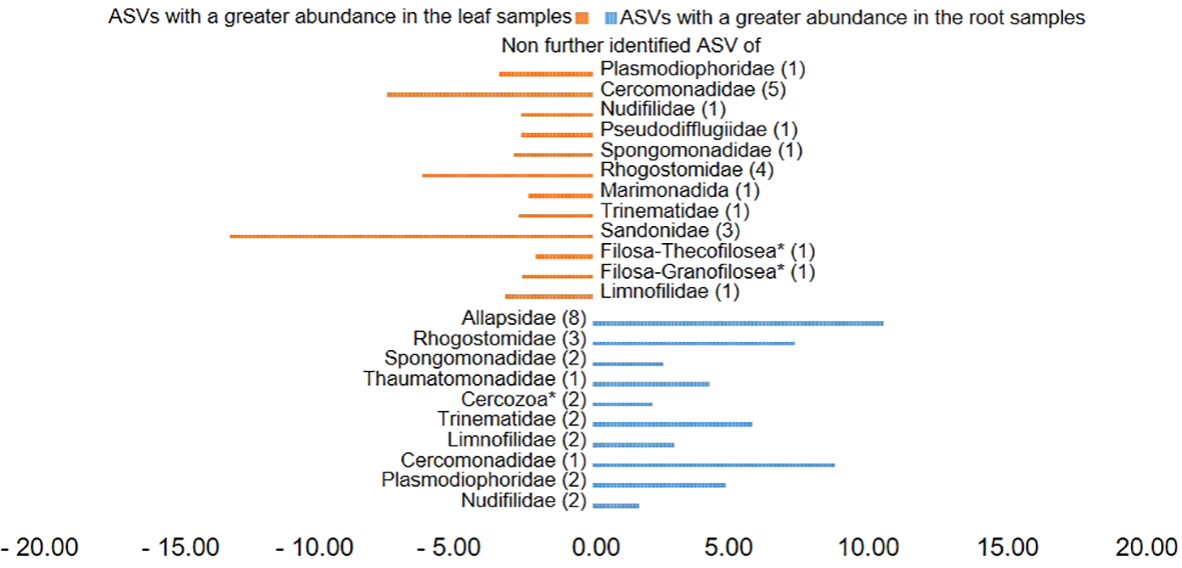
Divergent barplot with the ASVs (taxonomically not further determined than the family level) of the families that were significantly different in root and leaf samples. * Taxonomically not determined to family level. The number in brackets is the corresponding number of the ASV.

**Figure 5:**
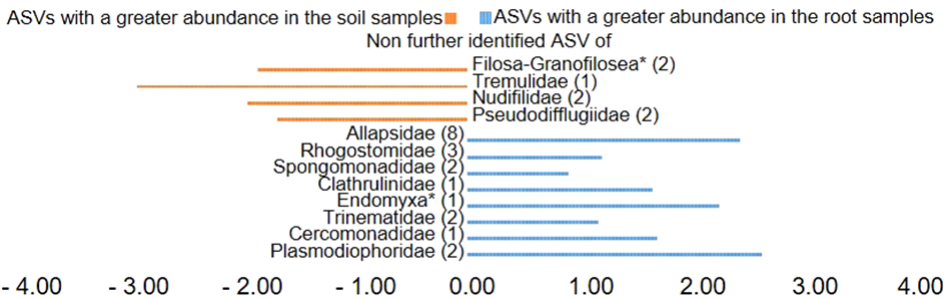
Divergent barplot with the ASVs (taxonomically not further determined than the family level) of the families that were significantly different in root and soil samples. * Taxonomically not determined to family level. The number in brackets is the corresponding number of the ASV.

**Figure 6:**
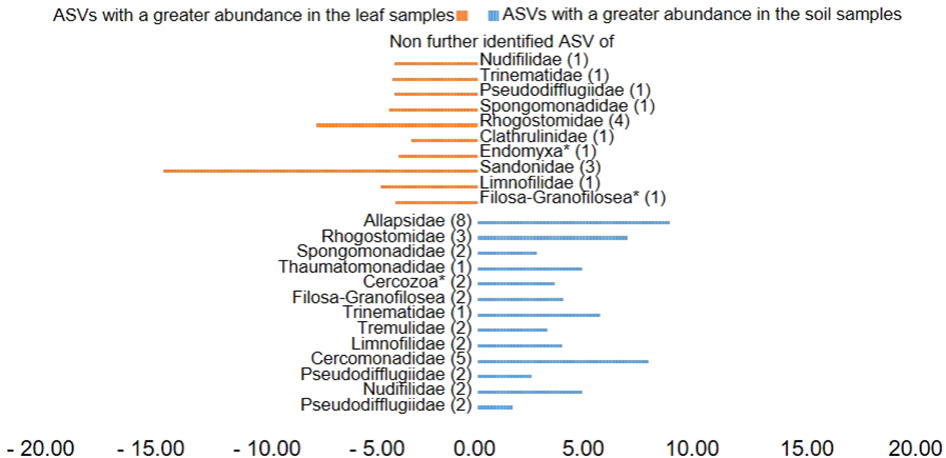
Divergent barplot with the ASVs (taxonomically not further determined than the family level) of the families that were significantly different in soil and leaf samples. * Taxonomically not determined to family level. The number in brackets is the corresponding number of the ASV.

Plotting of the clr value median difference between leaf and root samples (diff.btw plot of the ALDEx2 analysis) (**Figure 4**) showed an unidentified ASV of the family Sandonidae (3) as most abundant in leaf samples, and one of the Allapsidae (8) in root material. In parallel, the diff.btw plot of soil and root samples (**Figure 5**), showed an unidentified ASV of the family Tremulidae (1) as most abundant in the soil material, and Plasmodiophoridae (2) in the root material. The diff.btw plot of the ALDEx2 analysis of leaf and soil samples, showed the highest abundance in the leaf material, of an unidentified ASV of the family Sandonidae (3) and of an unidentified Allapsidae (8) in the soil material (**Figure 6**). The comparison of the ASVs with the biggest influence on the beta diversity from the arrows of **Figure 3** and the unidentified ASVs of the families from **Figures 4**–**6** showed two ASVs which were present in all graphics. The ASV from arrow 4, unidentified ASV of Allapsidae (8), was found in all ALDEx2 analyses as well as the ASV from arrow 8, unidentified ASV of Rhogostomidae (3).

## Discussion

The chemical analysis of the soils where the first and second seasons of spring barley seeds were sown showed similar values for the soil parameters, except for nitrate concentration (**Table 2**). In the second season soil nitrate levels were higher than in the first season. Protists are very sensitive to the various changes that occur in the soil. Krashevska et al. (2014) showed that the density of living cells of protists increased with higher N inputs into the soil, and a higher amount of nitrate in the soil of the second season of barley could have also led to an altered Cercozoa community. However, the analysis of the differences in beta diversity between the two seasons using two different algorithms did not reveal clear differences. The analysis with R-Studio (phyloseq 1.28.0) of the sampling material of season one and season two showed no significant differences between the sampling material at the different sampling points for the two seasons (**Supplementary Figure 1**), the analysis with QIIME2 (DEICODE) of the sampling material of season one and season two showed only significant differences (besides the comparisons of the leaf samples) between the different sampling materials at the different sampling points for the two seasons. Nevertheless, this project aimed to provide a robust analysis of the natural cercozoan community of spring barley, besides the naturally occurring changes between seasons and therefore we combined both seasons and analysed them as one.

The spring barley plants of both seasons of this study were exposed to very hot and dry summers (**Supplementary Table 5**). Dry conditions, especially on the leaf surface, created difficult circumstances for the cercozoan protists. For this reason, only the sequencing results of the root and soil material, and not those of leaf material, were pre-processed, as the former environments were less directly affected by drought. Due to such problems, researchers focus, in addition to high-throughput sequencing of leaf-associated protists, on other methods like microorganisms’ isolation, cloning or other plant compartments of important crops (Ploch et al., 2016; Flues et al., 2018; Rossmann et al., 2020). The unprocessed leaf material was analysed nonetheless, as it still best represents the leaves’ natural cercozoan composition in this analysis, and it focuses on future problems linked to the resilience of the plant’s natural cercozoan community during hot and dry summers resulting from man-made climate change.

Diversity analyses of the ten most dominant ASVs revealed a dominance of the family Sandonidae in all samples (**Figure 1**), at both flowering and ripening stages, except for the roots. Leaf surfaces in particular showed a high percentage of unidentified Sandonidae at the flowering (97.43%) and ripening stages (100%) (**Figure 1A**), while rhizosphere (**Figure 1C**) and bulk soils (**Figures 1D, E**) had lower percentages of those ASVs, although still dominant (30% for rhizosphere soil at flowering to 40% for bulk soils at the ripening stage and 60% after harvest). Interestingly, ASVs belonging to the Sandonidae family only remained on leaf surfaces by the ripening stage (**Figure 1A**). The lineages represented by these ASVs are likely highly adapted to drought stress on leaf surfaces, eventually forming cysts - a common trait among Cercozoa - giving them a significant advantage in changing climate conditions. Similarly, other Sandonidae ASVs were dominant in rhizosphere soils, within a particularly stable cercozoan community, with only a shift in abundance of the same ten most dominant ASVs from the flowering to the ripening stages (**Figure 1C**). Unfluctuating soil conditions, such as lower drought stress than on the leaves, and only a small concentration of exudates from older or dying roots at the ripening, allowed for a stable community, and selection of the most competitive lineages, further confirming the competitiveness of the Sandonidae ASVs. Common to biocrusts from grasslands and dunes (Roshan et al., 2021), Sandonidae exerted a strong dominance in bulk soils (**Figures 1D, E**). While there is a possibility of primer being more representative of Sandonidae 18S rRNA, Fiore-Donno et al. (2017) reported a 97% success rate in the analysis of a mock community with these primers.

In addition to Sandonidae, leaf samples also harboured unidentified ASVs of the families Allapsidae and Glissomonadida, confirming results by Ploch et al. (2016), who identified the genera *Sandona, Neoheteromita* and *Allapsa* in phyllosphere samples. The Allpasidae and Glissomondaida ASVs disappeared from leaf samples by the ripening stage, probably because those species were unable to endure the environmental conditions on the leaves (**Figure 1A**). Root samples were dominated by unidentified ASVs of the family Allapsidae (**Figure 1B**), known for inhabiting the roots and showing positive associations with plant genes involved in plant growth and development, and unidentified ASVs of the family Rhogostomidae were also associated with the cercozoan root community (Dumack et al., 2017; Rüger et al., 2023). Bulk soils before seeding, at flowering, at ripening and after harvest were also dominated by unidentified ASVs of the families Cercomonadidae, Rhogostomidae in addition to the aforementioned Sandonidae. Although slight variations in cercozoan composition were observed across different plant stages and sampling points, the top ten dominant ASVs remained mostly unchanged. Differences were raised through a strong prevalence of unidentified ASVs of the family Sandonidae in the leaf samples, unidentified ASVs of the family Allapsidae in the root samples, and mixed dominance in the soil samples. These findings were supported by the phylogenetic tree of the plant compartments - leaf, soil and roots (**Supplementary Figure 6**) – indicating the dominance of Sandonidae, Allapsidae and one Rhogostomidae (2) in the top ten unidentified ASVs, along with ASVs from Glissomonadida (3) and Cercomonadidae (1). In this context, it is important to note that protists possess varying quantities of 18S rRNA gene copies (Heywood et al., 2011). The variation in the number of 18S rRNA gene copies, potentially influenced by factors such as the environment, genome size, and cell size, may result in misrepresentation and overestimation of their presence, thereby compromising the accuracy of the results (Prokopowich et al., 2003; Zhu et al., 2005; Gong and Marchetti, 2019; Pan et al., 2022).

Alpha diversity analyses further confirmed compartment-specific cercozoan communities of leaf, root and soil, independent of the analysed index, with a clear separation between leaf, soil and root samples. No significant differences in alpha diversity were found between the different leaf, soil and root samples (**Supplementary Tables 1-3**). This means that the alpha diversity of the cercozoan community of spring barley is independent of the plant stage and was primarily influenced by the sampling material. The very low alpha diversity of the leaf samples can be explained by the very dry, hot summer and the harsh conditions aboveground, which reduced the richness of the cercozoan community from the leaves. In contrast, the very high alpha diversity observed in the soil can be explained by more favourable underground conditions concerning water availability and temperature. The alpha diversity of the root sampling material, which was far lower than the alpha diversity of the soil samples but a little bit higher than that of the leaf samples, can be explained by the influences and repelling exudates of the heat-suffering plant.

Beta diversity analyses initially confirmed previous results, with a clear separation of the diversity of the leaf, the root and the soil sampling material and only some overlaps (**Figure 3**). However, PERMANOVA analysis showed some interesting significant differences (**Supplementary Table 4**). The community of the leaves at the flowering stage was significantly different from the community of the leaves at the ripening stage: harsh environmental conditions, such as drought and high solar radiation, and the dying leaves, which were yellow at the second sampling, created a niche for highly competitive Cercozoa. Given the previously observed strong shift of the ten most abundant phyllosphere cercozoan ASVs (**Figure 1A**), a significant difference in community compositions between these two stages, although not shown before, is not surprising. Furthermore, the leaf community composition at the flowering stage was significantly different in comparison to that of all the other communities of root and soil material, except for bulk soil. These significant differences support alpha diversity analysis results and the assumption of plant-compartment-specific cercozoan communities. The lack of significant differences in comparison to the bulk soil samples was more surprising. During germination, the first contact of the leaves with the bulk soil probably influenced the cercozoan composition of the leaf at flowering, explaining converging beta diversity. Moreover, wind dispersal and rainfalls also impact beta diversity (Genitsaris et al., 2014; Jauss et al., 2021). Nevertheless, environmental conditions changed the cercozoan phyllosphere community from the flowering to the ripening stage: at the ripening stage, the community was significantly different from all soil and root samples, showing a clear separation between the plant compartments again. Rhizosphere soil community was significantly different at flowering from that at ripening; community differences were most likely due to the plant’s senescent state at ripening. Rhizosphere soil communities were not significantly different to bulk soil communities, and bulk soil communities did not show significant differences between each other but were significantly different to the root communities. This further confirms the plant’s specific compartments, explaining why beta diversity did not differ between samples, except for really strong environmental (and subsequent plant) influences. The root communities showed no significant differences between the flowering and ripening stages, which was unexpected compared to the previous results mentioned in this paragraph and influence of the dying plant. Clearly, the exudates of the dying plant and the reduction of the dominating unidentified species of the family Allapsidae did not influence the beta diversity enough. The beta diversity was strongly driven by some single ASVs. These belonged to the unidentified species of the families Sandonidae, Allapsidae and Rhogostomidae. While unidentified ASVs of the family Allapsidae dominated the root samples, the unidentified ASVs of the family Sandonidae were present in a lot of samples and dominated the leaf communities. These drivers probably influenced the formation of the different plant compartments.

ALDEx2 analyses considered plant compartments and their specific cercozoan communities, and how these differed between leaf, root and soil. Comparison of leaf and root communities showed higher abundance than the mean of an unidentified Sandonidae (3) ASV in the leaf samples and an unidentified ASV of the Allapsidae (8) family in the root samples (**Figure 4**). These ASVs had the highest diff.btw values, and not unexpected given previously discussed results. The second highest diff.btw value in both compartments was shown by two different unidentified ASVs of the Cercomonadidae (5 and 1) family. They seem to be more generalist and occur in both compartments in higher abundances. There were fewer shifts in the comparison of soil and root communities than there were in the comparisons with the leaves (**Figure 5**). In the beta-diversity plot were also more overlapping points between those two compartments (**Figure 3**). The unidentified ASV of the family Tremulidae (1) had a higher abundance in the soil samples. Although not much is known about this family, the genus *Tremula* has been shown to survive dry soil conditions (Howe et al., 2011), and the protist *Tremula longifila*, usually cultured with eukaryotic prey, can also survive with bacterial prey in culture, indicating a functional flexibility under harsh environmental conditions. The unidentified ASVs of the families Plasmodiophoridae (2) and Allapsidae (8) were differentially abundant in the roots. Members of the family Plasmodiophoridae are not only plant parasites with strong differences within the species (Bulmana et al., 2001), but also well-known plant pathogens. Plasmodiophorids are also parasites and symbionts of stramenopiles (Neuhauser et al., 2014), commonly found in soils (Bates et al., 2013; Dupont et al., 2016). Their presence is therefore unsurprising. In line with the diversity analyses discussed above, unidentified ASVs of the Allapsidae family dominated the root compartment; when comparing the leaf and soil communities, the unidentified Sandonidae (3) ASV had higher abundance in the leaf samples, and the unidentified Allapsidae (8) family in the soil samples (**Figure 6**). The unidentified ASV of the Rhogostomidae (4) family also showed a high diff.btw value in the leaf samples. Previously found on leaves (Dumack et al., 2017), Rhogostomidae are believed to be able to withstand drier conditions thanks to their theca and resting stages (Belar, 1921). The unidentified ASV of the Cercomonadidae (5) family showed a high diff.btw value in the soil samples, similar to the comparison between leaf and root compartments. To better understand the ecological significance of these ASVs, which was not possible in this study due to the poor taxonomic resolution only up to the family level, future studies should also try to improve the taxonomic resolution. For this purpose, attempts should be made to isolate the Cercozoa species from this habitats or to find out more about their function by sequencing metagenomes, as has already been done by Thompson et al. (2020) in an Antarctic soil ecosystem or Oliverio et al. (2020) in below ground systems. However, differential abundance analysis showed greater overlaps between the compartments than previous analyses. These results were confirmed by the phylogenetic tree of the plant compartments (**Supplementary Figure 6**). The phylogenetic tree of the ten most dominant on family level identified ASVs showed that each of the ASVs occurred in at least one sample of the root compartment. Furthermore, the ASVs occurred in at least one sample from one of the other compartments or in samples of all compartments. Although the phylogenetic tree is dominated by the occurrence of the unidentified ASVs in the soil samples, several connecting points between the three plant compartments are pointed out.

## Conclusion

*Hordeum vulgare* is one of the most economically important crops, and protists are an important part of the plant holobiome and influence plant growth and pathogen pressure. The natural composition of the cercozoan eukaryome is an important tool for understanding plant performance. The sequencing results showed a clear separation between the compositions of the leaf, soil, and root samples, and a dominance of unidentified ASVs of the family Sandonidae in leaf samples, the family Allapsidae in root samples and a mixed dominance of unidentified ASVs of the families Sandonidae, Allapsidae, Cercomonadidae and Rhogostomidae in soil samples. It can be concluded that the cercozoan diversity of spring barley was highly determined by the plant compartment and not by the growing stage of the plant. Only the leaf material at flowering and ripening stages showed significant differences in the cercozoan composition, which was attributable to the strong environmental influences. However, the cercozoan community composition of rhizosphere soil, bulk soil and roots did not change significantly during plant growth. Based on those results, further analyses of the cercozoan community of other important crops could confirm the influence of the plant compartment. A more general understanding of the cercozoan community composition on the leaf, root and soil compartments of different crop plants together with analysis of the feeding behaviour of cercozoans on the crop plants could enable future recommendations for management actions in organic farming for improving plant growth and mitigating pathogen pressure.

## Data availability statement

The datasets presented in this study can be found in online repositories. The names of the repository/repositories and accession number(s) can be found below: https://www.ncbi.nlm.nih.gov/sra/PRJNA1043645, PRJNA1043645.

## Author contributions

JS: Conceptualization, Data curation, Funding acquisition, Investigation, Methodology, Writing – original draft. SR: Conceptualization, Investigation, Methodology, Supervision, Validation, Writing – review & editing. SQ-Q: Conceptualization, Investigation, Methodology, Writing – review & editing. RG-P: Conceptualization, Investigation, Methodology, Writing – review & editing. BS: Investigation, Methodology, Writing – review & editing. AÖ: Conceptualization, Funding acquisition, Investigation, Supervision, Writing – review & editing. SS: Conceptualization, Funding acquisition, Investigation, Methodology, Project administration, Supervision, Writing – review & editing.
