# Supplementary material for: Cercozoan diversity of spring barley grown in the field is strongly plant compartment specific
Source: Front Microbiomes. 2024 Feb 8;3:1352566. doi: 10.3389/frmbi.2024.1352566 (PMC12993544; doi:10.3389/frmbi.2024.1352566)
Supplement: Supplementary file 1 [file DataSheet_1.pdf]

Supplementary Material

Cercozoan diversity of spring barley grown in the field is strongly plant compartment specific

Julia Sacharow[1*], Stefan Ratering[1], Santiago Quiroga[1], Rita Geißler-Plaum[1], Bellinda Schneider[1], Alessandra Österreicher Cunha-Dupont[1] and Sylvia Schnell[1]

Professorship of General and Soil Microbiology, Institute of Applied Microbiology, IFZ, Justus-Liebig-University Giessen, Giessen, Germany [1]

[*] **Correspondence:**

Julia Sacharow

Julia.Sacharow@en.uni-giessen.de

Submission supplementary material tables

Table S1: Alpha diversity – *p*-values of the pairwise comparisons of the cercozoan communities from the sampling material roots, soil and leaves at the sampling points before seeding, at flowering, at ripening and after harvest using Wilcoxon rank sum test with Holm correction (observed richness). * *p*-value < 0.05.

	Leaves flowering	Leaves ripening	Bulk soil before seeding	Bulk soil flowering	Rhizosphere soil flowering	Bulk soil ripening	Rhizosphere soil ripening	Bulk soil after harvesting	Roots flowering
Leaves ripening	1.000	-	-	-	-	-	-	-	-
Bulk soil before seeding	0.006*	0.027*	-	-	-	-	-	-	-
Bulk soil flowering	0.006*	0.027*	1.000	-	-	-	-	-	-
Rhizosphere soil flowering	0.000*	0.008*	1.000	1.000	-	-	-	-	-
Bulk soil ripening	0.006*	0.027*	1.000	1.000	1.000	-	-	-	-
Rhizosphere soil ripening	0.084	0.150	1.000	1.000	1.000	1.000	-	-	-
Bulk soil after harvesting	0.027*	0.027*	1.000	1.000	1.000	1.000	1.000	-	-
Roots flowering	0.006*	0.027*	0.006*	0.006*	0.009*	0.006*	0.084	0.027*	-
Roots ripening	0.006*	0.027*	0.006*	0.022*	0.006*	0.006*	0.084	0.027*	1.000

Table S2: Alpha diversity - *p*-values of the pairwise comparisons of the cercozoan communities from the sampling material roots, soil and leaves at the sampling points before seeding, at flowering, at ripening and after the harvest using Wilcoxon rank sum test with Holm correction (Shannon richness). * *p*-value < 0.05.

	Leaves flowering	Leaves ripening	Bulk soil before seeding	Bulk soil flowering	Rhizosphere soil flowering	Bulk soil ripening	Rhizosphere soil ripening	Bulk soil after harvesting	Roots flowering
Leaves ripening	1.000	-	-	-	-	-	-	-	-
Bulk soil before seeding	0.006*	0.006*	-	-	-	-	-	-	-
Bulk soil flowering	0.006*	0.006*	1.000	-	-	-	-	-	-
Rhizosphere soil flowering	0.000*	0.000*	1.000	1.000	-	-	-	-	-
Bulk soil ripening	0.006*	0.006*	1.000	1.000	1.000	-	-	-	-
Rhizosphere soil ripening	0.084	0.084	0.727	0.480	1.000	1.000	-	-	-
Bulk soil after harvesting	0.006*	0.006*	1.000	1.000	1.000	1.000	0.480	-	-
Roots flowering	0.006*	0.007*	0.006*	0.006*	0.000*	0.006*	0.084	0.006*	-
Roots ripening	0.006*	0.007*	0.006*	0.006*	0.000*	0.006*	0.084	0.006*	1.000

Table S3: Alpha diversity - *p*-values of the pairwise comparisons of the cercozoan communities from the sampling material roots, soil and leaves at the sampling points before seeding, at flowering, at ripening and after the harvest using Wilcoxon rank sum test with Holm correction (Fischer richness). * *p*-value < 0.05.

	Leaves flowering	Leaves ripening	Bulk soil before seeding	Bulk soil flowering	Rhizosphere soil flowering	Bulk soil ripening	Rhizosphere soil ripening	Bulk soil after harvesting	Roots flowering
Leaves ripening	1.000	-	-	-	-	-	-	-	-
Bulk soil before seeding	0.006*	0.006*	-	-	-	-	-	-	-
Bulk soil flowering	0.006*	0.006*	1.000	-	-	-	-	-	-
Rhizosphere soil flowering	0.000*	0.000*	1.000	1.000	-	-	-	-	-
Bulk soil ripening	0.006*	0.006*	1.000	1.000	1.000	-	-	-	-
Rhizosphere soil ripening	0.084	0.084	1.000	1.000	1.000	1.000	-	-	-
Bulk soil after harvesting	0.006*	0.006*	1.000	1.000	1.000	1.000	1.000	-	-
Roots flowering	0.006*	0.006*	0.006*	0.006*	0.001*	0.006*	0.084	0.006*	-
Roots ripening	0.006*	0.006*	0.006*	0.006*	0.002*	0.006*	0.084	0.006*	1.000

Table S4: Beta diversity - *p*-values of the pairwise comparisons of the cercozoan communities from the sampling material roots, soil and leaves at the sampling points before seeding, at flowering, at ripening and after the harvest using PERMANOVA test with Benjamini-Hochberg correction. * *p*-value < 0.05.

	Leaves flowering	Leaves ripening	Bulk soil before seeding	Bulk soil flowering	Rhizosphere soil flowering	Bulk soil ripening	Rhizosphere soil ripening	Bulk soil after harvesting	Roots flowering
Leaves ripening	0.030*	-	-	-	-	-	-	-	-
Bulk soil before seeding	0.137	0.001*	-	-	-	-	-	-	-
Bulk soil flowering	0.102	0.002*	0.969	-	-	-	-	-	-
Rhizosphere soil flowering	0.047*	0.002*	0.305	0.376	-	-	-	-	-
Bulk soil ripening	0.103	0.001*	0.617	0.608	0.123	-	-	-	-
Rhizosphere soil ripening	0.027*	0.002*	0.207	0.190	0.026*	0.237	-	-	-
Bulk soil after harvesting	0.101	0.001*	0.584	0.522	0.077	0.658	0.234	-	-
Roots flowering	0.003*	0.001*	0.001*	0.001*	0.001*	0.001*	0.005*	0.001*	-
Roots ripening	0.001*	0.001*	0.608	0.001*	0.001*	0.001*	0.005*	0.001*	0.532

Table S5: Weather data of 2021 and 2022 for the area of the Gladbacher Hof.

**2021**	Air temperature $\bar{X}$ in °C	Air temperature min. in °C	Air temperature max. in °C	Air humidity $\bar{X}$ in %
January	1.7	- 6.1	11.2	90
February	2.4	- 11.8	19.7	85
March	5.7	- 6.0	25.7	75
April	6.9	- 3.2	23.7	70
May	11.4	- 1.5	28.7	73
June	19.9	6.6	34.0	74
July	18.4	9.3	28.6	79
August	16.9	7.9	30.4	82
September	15.4	4.1	28.0	83
October	9.7	- 0.9	21.9	88
November	4.9	- 4.8	15.9	93
December	3.9	- 7.6	15.1	92
**2022**				
January	3.3	- 4.1	12.8	91
February	5.4	- 5.3	14.5	80
March	6.1	- 5.3	21.2	64
April	9.0	- 4.0	24.2	70
May	15.5	2.9	29.6	70
June	19.1	4.1	35.9	70
July	20.2	7.3	37.0	63
August	21.8	8.1	37.1	58
September	14.1	2.8	31.4	81
October	12.4	0.1	23.8	90
November	7.5	- 0.6	17.8	90
December	2.7	- 11.5	18.3	91

Submission supplementary material figures

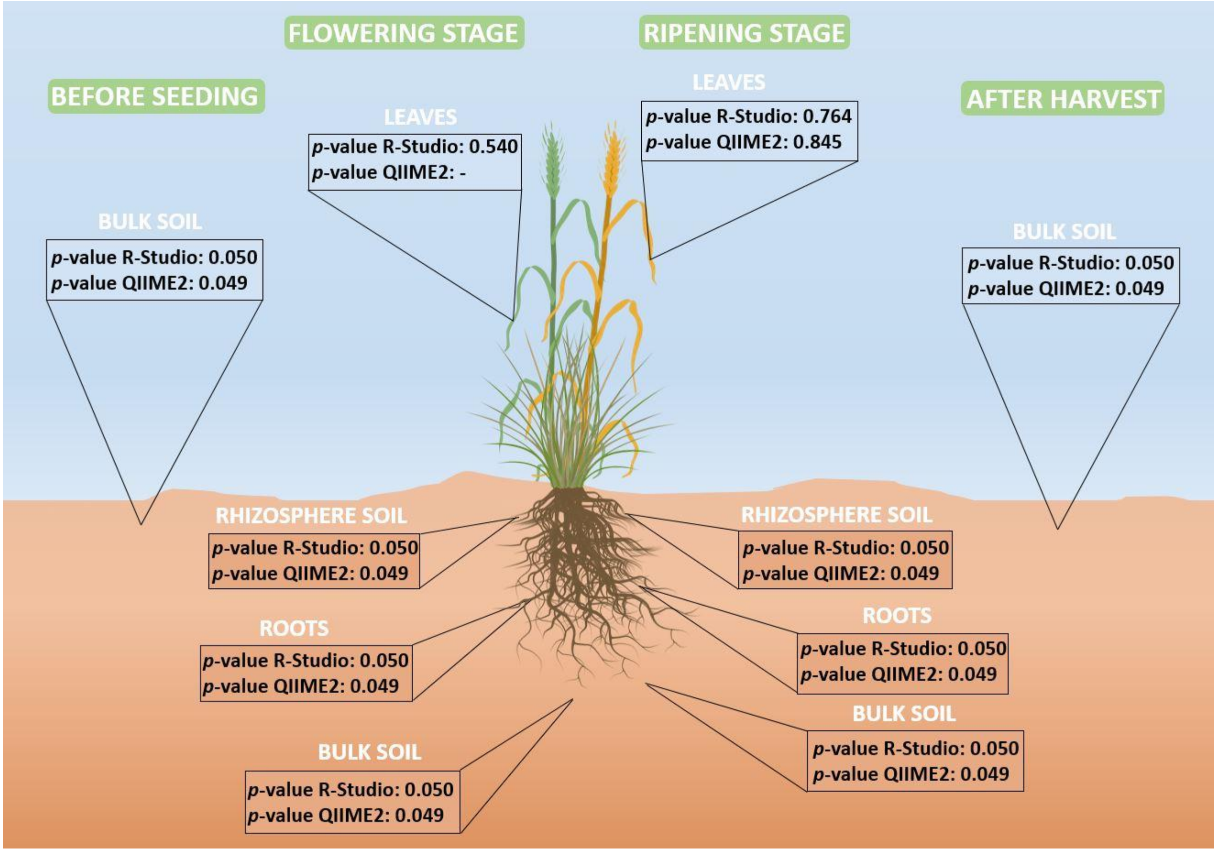

Figure S1: Benjamini-Hochberg adjusted *p*-values (beta diversity) of the pairwise comparison from the sampling material bulk soil, leaves, rhizosphere soil and roots from seasons one and two of spring barley at the sampling points before seeding, at flowering, at ripening and after the harvest. Analyses were performed with R-Studio and QIIME 2. – *p*-value could not be calculated.

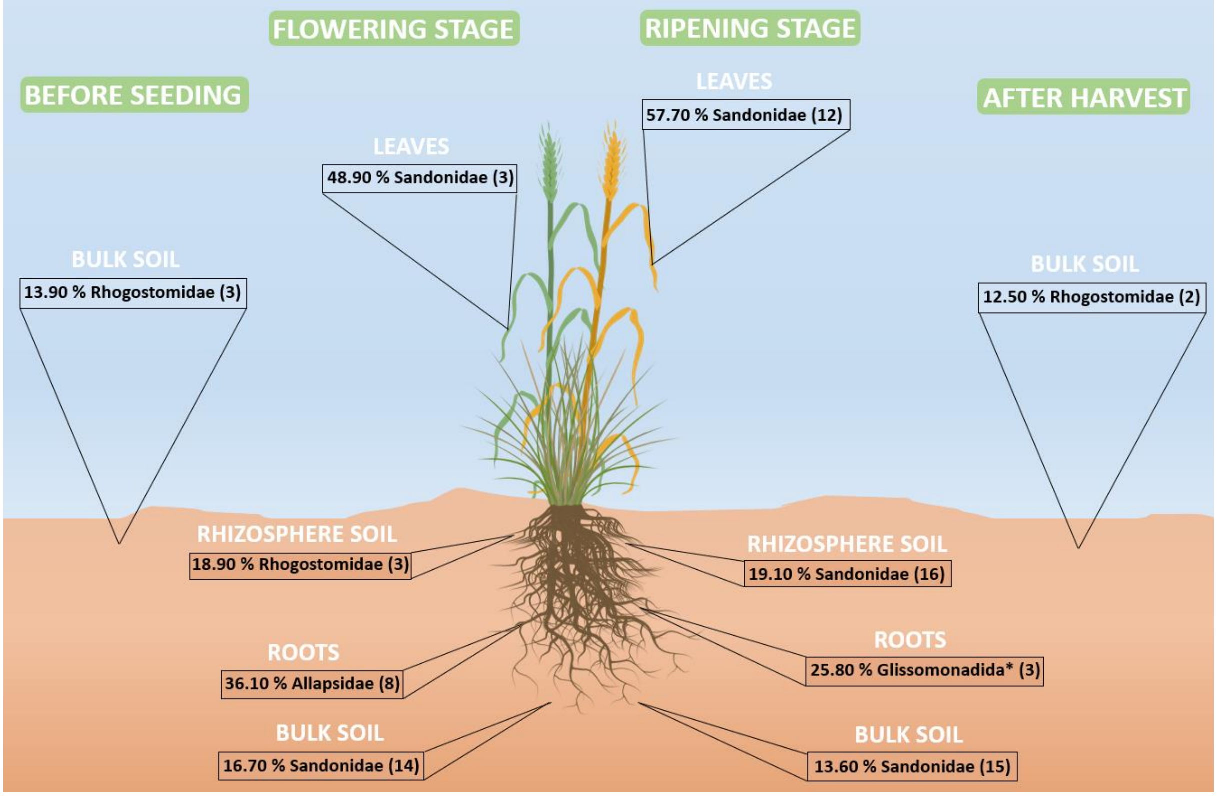

Figure S2: Most abundant (taxonomically not further determined than the family level) ASVs of cercozoan families of leaf, root, rhizosphere soil and bulk soil samples depending on the sampling point before seeding, at flowering, at ripening and after harvesting. * Taxonomically not determined to family level. The number in brackets is the corresponding number of the ASVs.

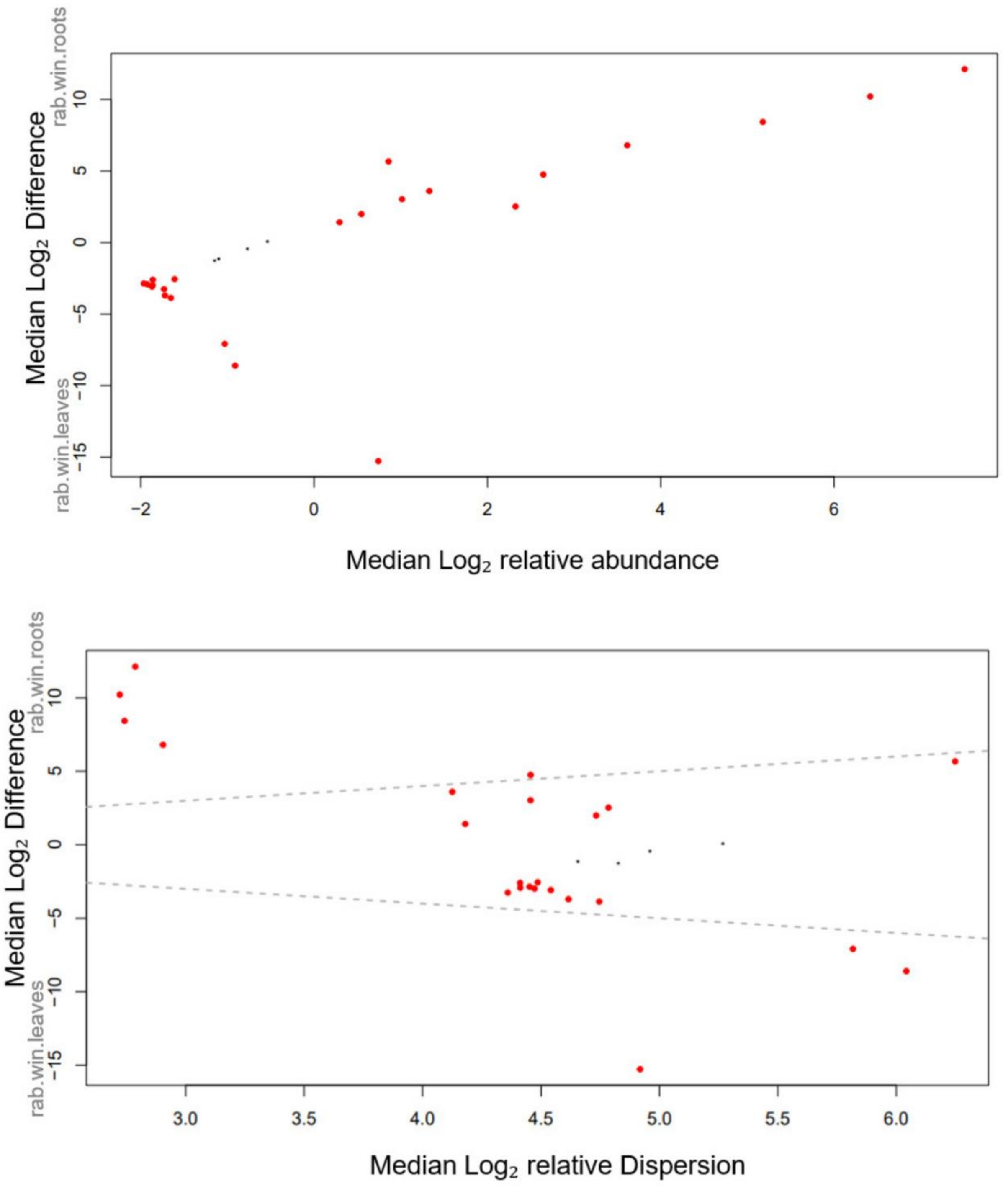

Figure S3: MW (above) and MA (below) plots. Roots vs leaves distribution of ASVs significantly different (red dots) from the sample mean by Wilcox test with Benjamini–Hochberg correction. ASVs with higher abundance than the mean in root samples have positive diff.btw values, while ASVs with higher abundance than the mean in leaf samples have negative values.

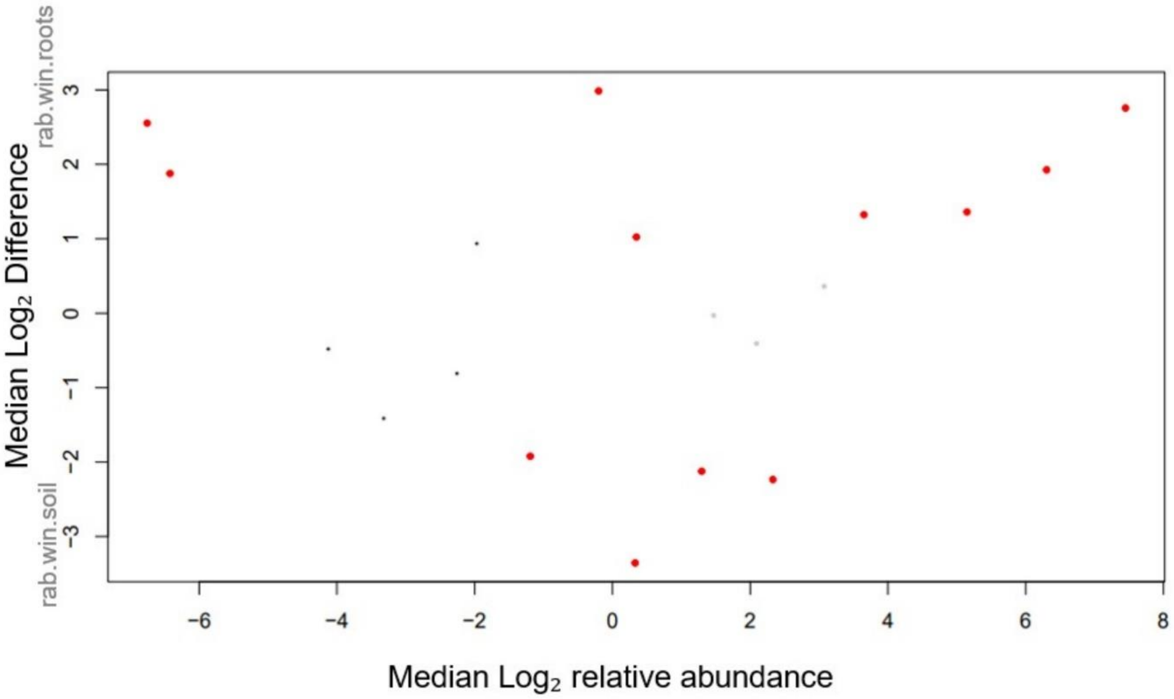

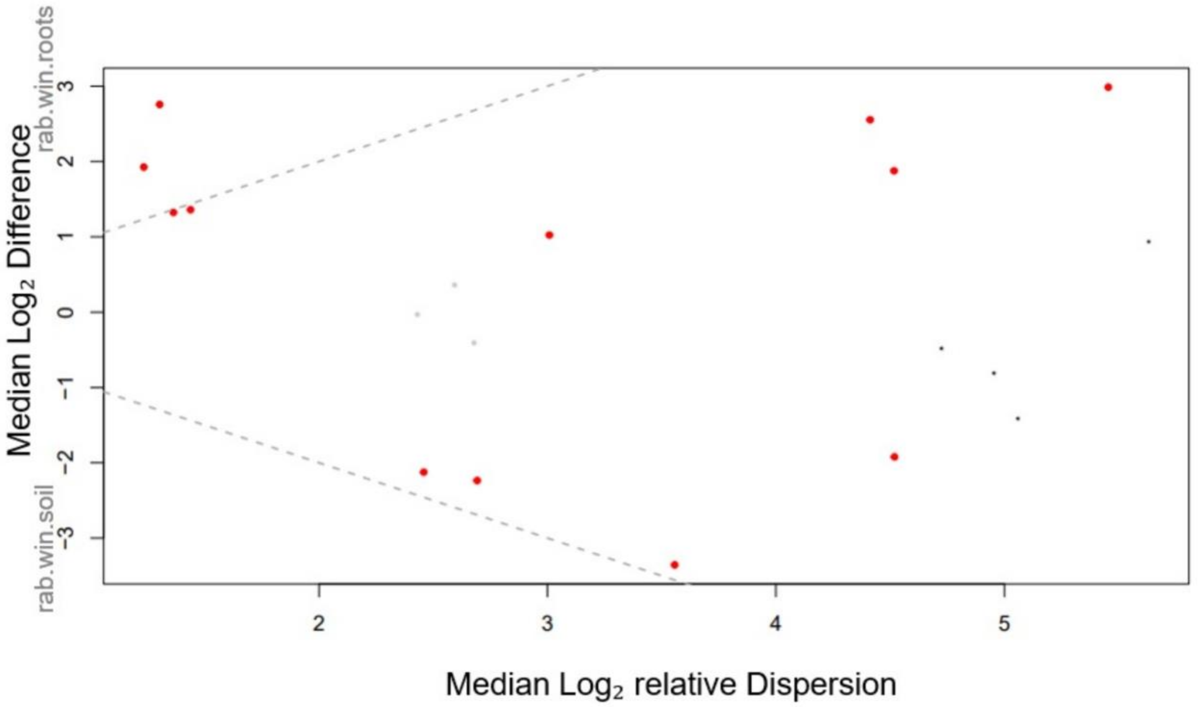

Figure S4: MW (above) and MA (below) plots. Roots vs. soil distribution of ASVs significantly different (red dots) from the sample mean by Wilcox test with Benjamini–Hochberg correction. ASVs with higher abundance than the mean in root samples have positive diff.btw values, while ASVs with higher abundance than the mean in soil samples have negative values.

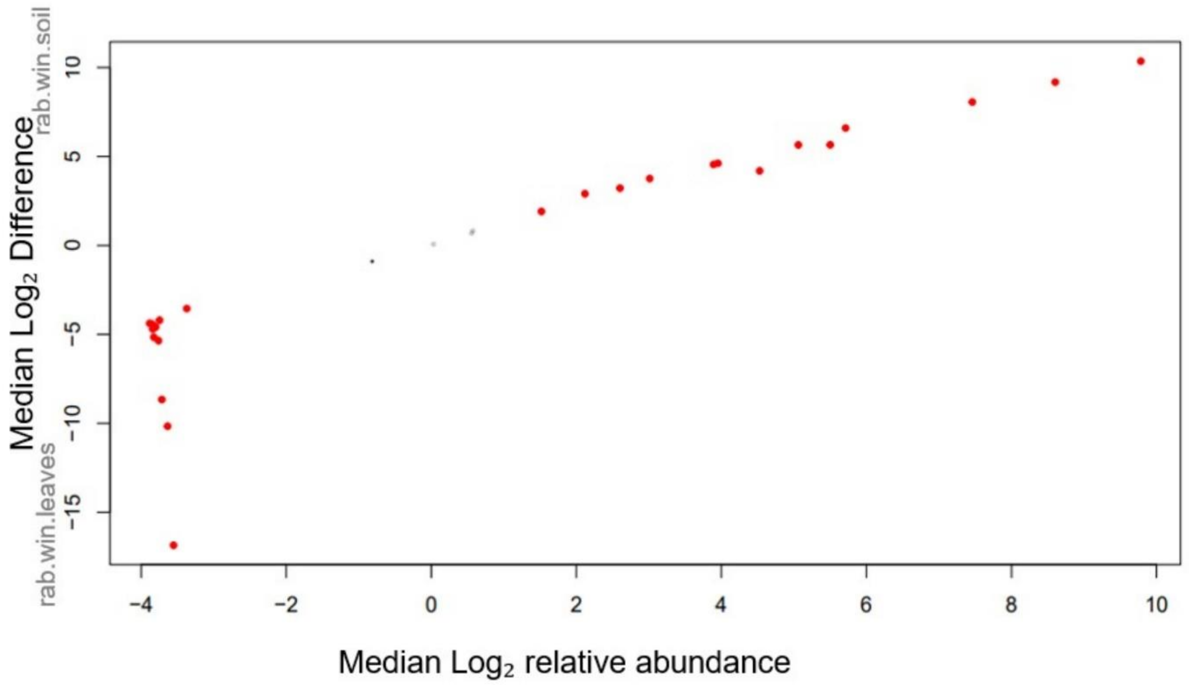

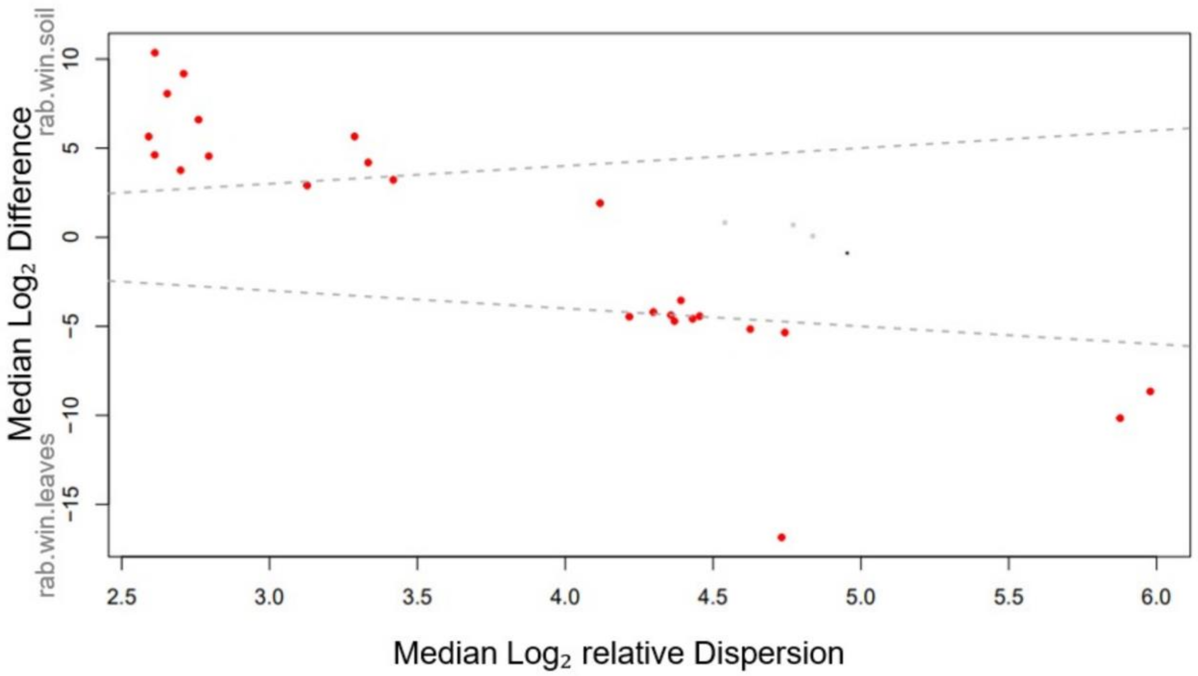

Figure S5: MW (above) and MA (below) plots. Soil vs. leaves distribution of ASVs significantly different (red dots) from the sample mean by Wilcox test with Benjamini–Hochberg correction. ASVs with higher abundance than the mean in soil samples have positive diff.btw values, while ASVs with higher abundance than the mean in leaf samples have negative values.

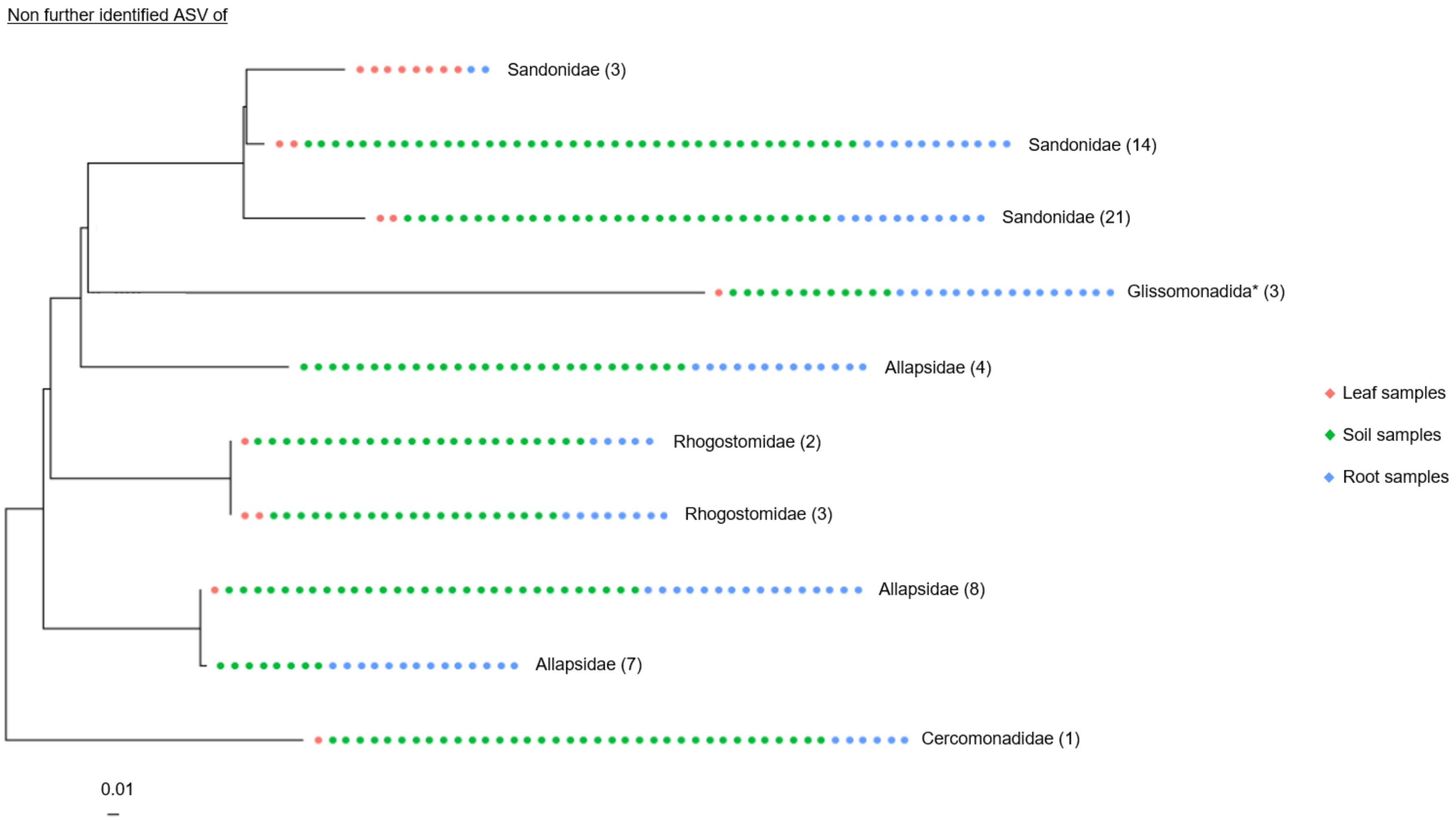

Figure S6: Phylogenetic tree (maximum likelihood) of the ten most dominant taxonomically on family level identified ASVs of the leaf, soil and root compartments of the spring barley with markers, one for each leaf sample (red), soil sample (green) and root sample (blue) in which the ASV was observed. The number in brackets is the corresponding number of the ASVs.
